# Preference Mining Using Neighborhood Rough Set Model on Two Universes

**DOI:** 10.1155/2016/6975458

**Published:** 2016-12-04

**Authors:** Kai Zeng

**Affiliations:** Faculty of Information Engineering, Guizhou Institute of Technology, No. 1 Caiguan Road, Guiyang 550003, China

## Abstract

Preference mining plays an important role in e-commerce and video websites for enhancing user satisfaction and loyalty. Some classical methods are not available for the cold-start problem when the user or the item is new. In this paper, we propose a new model, called parametric neighborhood rough set on two universes (NRSTU), to describe the user and item data structures. Furthermore, the neighborhood lower approximation operator is used for defining the preference rules. Then, we provide the means for recommending items to users by using these rules. Finally, we give an experimental example to show the details of NRSTU-based preference mining for cold-start problem. The parameters of the model are also discussed. The experimental results show that the proposed method presents an effective solution for preference mining. In particular, NRSTU improves the recommendation accuracy by about 19% compared to the traditional method.

## 1. Introduction

In recent years, electronic retailers and content providers offer a huge selection of products. It makes the users confused in making decisions among various kinds of products (called items in this paper), such as books, music, and movies. Matching consumers with most appropriate items is important in enhancing user satisfaction and loyalty.

Many researchers have proposed different solutions to discover the preference relations between user and items. The most popular such technology is collaborative filtering (CF) [1]. There are two primary disciplines of CF: the neighborhood approach and latent factor models. A user-based neighborhood [2] approach evaluates the preference of a user by analyzing the historical rating data of neighbors who have similar taste to this user. The similar taste is usually interpreted that these users have rated most of the same items. Items that the neighbors like are then recommended to this user, as he/she will probably also like them. Latent factor models [3] built a two-dimensional matrix to describe the relation about users and items. The elements in the matrix are the ratings given by users. Thus the issue about preference mining translates to the issue about matrix completion. However, the classical CF-based method cannot work when it meets a new user who never rates any item [4]. As a matter of fact, there even are some long-time users who have never rated any of items. It is difficult to recommend an item since there is not any historical rating information about these users to facilitate personalized recommendation. The key reason is that the CF method only relies on the historical rating data. This problem can be called cold-start which is the intrinsic limitation of CF [4].

To deal with the cold-start problem [5], Schein et al. [6] developed a combined method for recommending items that no one has yet rated. Bobadilla et al. [7] proposed improved collaborative filtering to mitigate the new user cold-start problem. The content-based filtering methods [8, 9] are also studied for the cold-start problem. However, most of the researchers have addressed the cold-start problem where either the user or the item is new. The situation with both new user and new item has seldom been considered [4]. To cope with cold-start problem when the users and items are new, Min and Zhu [4] pioneered a cold-start recommendation approach for the new situation based rough set on two-universe model (RSTU). First, the authors considered the features of users' and items', such as age, gender, and price, to form the equivalence-based information granules. Then they use the definition of approximation operator in rough set theory for generating association rules between users and items. This granule-based recommendation model does not only rely on the rating data. It can work on better ways for dealing with the cold-start problem. However, some problems in [4] still need to be studied. For example, the equivalence-based information granule is only suitable for dealing with nominal data, such as male or female, good or bad. If the attributes are numerical, such as the price of the items, we have to adopt discretization technique to transform the nonnominal to the nominal, which would bring loss of information inevitably. It is obviously unreasonable to measure similarity or dissimilarity with Euclidean distance as to categorical attributes in numerical methods. Furthermore, the usual scoring method contains 5 scales, while the authors. [4] only consider whether or not a user has rated a movie. That is, a user will be labeled “like” if he has rated an item. It means that the user, who gives an item low mark, could be regarded as an admirer of the item. In fact, he takes dislike to the item because of the low-scoring. The key reason is that there is no evaluation about the user's rating baseline in [4]. This is also indistinguishable between positive rules and negative rules as the results.

Thus, so far, little work in this field has succeeded in holding the promise of personalized recommendation because of the following problems:

1. cold-start problems;
2. loss of information by discretization;
3. no distinction between positive rules and negative rules.

Aiming at above problems, the contribution of this paper includes the following: (1) we construct the parametric neighborhood rough sets model on two universes. Users and items are described through neighborhood granules. It can overcome the loss of information by the discretization of the data. (2) The rating table is divided into positive mapping and negative mapping based by using the rating baseline. The neighborhood lower approximation operator is used for defining the preference rules. Then, we can get the positive preference rules which means “like.” Some negative preference rules are also mined to be seen as “dislike.” (3) The method based on the neighborhood preference rules is proposed. The cold-start problem can be solved well. The experimental results show that our model presents an effective solution for preference mining.

The paper is organized as follows: Section 2 briefly reviews the related work about cold-start issue and rough set theory on two universes. In Section 3, some basic concepts about neighborhood rough set model on single universe and granular computing-based preference mining are briefly reviewed. In Section 4, the data model and the method about baseline evaluation are investigated. In Section 5, we propose the parametric neighborhood rough set model on two universes (NRSTU).  Section 6 shows the applications of NRSTU for preference rules mining and recommendation. Numeric experiments are reported in Section 7. Finally, Section 8 concludes the paper.

## 2. Related Work

### 2.1. The Cold-Start Issue

In a real recommender system, users usually present their interest by five numeric scores [10]. CF-based approach is based on the assumption that the users who have similar rating-behaviors are grouped together to help each other make a choice among the potential items. However, CF-based method is unavailable because of the cold-start problem. It occurs when the recommender system is short of ratings. We can distinguish three kinds of cold-start problems: only new user, only new item, and both of new user and new item [7].

#### 2.1.1. New User [11–13]

New user means we face the user who never rates any item. It is hard for a new user to obtain the preferred items since no available usage information can be employed in personalized recommendation [10]. The improved CF-based method such as CF-content or CF-demographic is the common strategy to tackle the new user problem [7]. For example, Braunhofer aimed at solving cold-start problem by using various contextually tagged rating datasets [14]. Leung et al. proposed a new content-based hybrid approach that makes use of cross-level association rules to integrate content information about domains items [15]. Ahn presented a new heuristic similarity measure named PIP that focuses on improving recommendation performance under new user condition [16]. All the former approaches are based on the assumption that the users are new. As a matter of fact, there could also be lots of new items in a real recommender system.

#### 2.1.2. New Item [17, 18]

The new item problem arises due to the fact that the new items have no initial ratings. New items are not likely to be recommended since there is not any rating information to facilitate personalized recommendation. Simplest solution is to encourage the motivated users who are responsible for rating each new item in the system [7]. In addition, the authors [19] investigated the value of even a few ratings in regard to predictive power. Cremonesi et al. presented two different approaches for building hybrid collaborative content recommender systems to overcome the new item issue [5]. Elbadrawy and Karypis proposed a feature-based similarity model for top-n recommendation of new items [20]. In a word, the new item problem [21] is more often addressed.

#### 2.1.3. New User and New Item

The new user or new item problem is addressed individually. However, the situation with both new user and new item has seldom been considered. It is very difficult to recommend a new item to a new user since the historical data of the current user and item is unknown. Rough set model so far is the best way to deal with this situation. Min and Zhu studied the cold-start problem by using the rough set model on two universes. They provide a means for describing users and items through information granules, a means for generating association rules between users and items, and a means for recommending items to users using these rules [4]. Min and Zhu explained the concept about granular association rule in detail [22]. Then, some rough set based methods are proposed to solve the new user and new item problem by extending Min work [23, 24].

### 2.2. Rough Set Model on Two Universes

The rough sets theory [25], proposed by Pawlak in 1982, is a powerful mathematical method for the study of incomplete or imprecise information. This theory has been successfully applied to many fields, such as data mining, decision making, pattern recognizing, machine learning, and intelligent controlling [26–29].

The Pawlak approximation operators are defined by an equivalence relation on the universe. The equivalence relation forms a partition of the universe. Partition or equivalent relation is still restrictive for many applications. To address this problem, Lin firstly proposed the theory of neighborhood system [30–32]. The idea about neighborhoods of tolerance is pointed out by Professor Lin. Neighborhood theory can be seen as a breakthrough of rough set. Professor Zhu studied the covering-based rough set [33, 34]. He gives the definition of smallest covering. It is a key issue to reduce the redundant information in data mining. Zhu investigated the relationship among basic concepts in covering-based rough sets. This excellent research helps us have a better understanding of covering-based rough set. Neighborhood rough set and cover-based rough set are all meaningful extensions to equivalent relation.

Most of the researches have been conducted on the assumption of the same universe [26–29]. However, two-universe model is more appropriate for the preference mining problem. In general, for a certain user, he or she may grade many items. Meantime, it also could include many items rated by some users in a real recommender system. Then an effective method to describe this problem is using two different universes. One of the universes is the set of all users. Another is the set of all the items. Some results have been generated in the rough sets theory on two universes. In [35], the authors gave a general framework of the two-universe based rough sets model. Shen and Wang defined a variable precision rough sets model based on classical Pawlak model, in which the lower approximation and the upper approximation were generalized to two universes [36]. The concept of the probabilistic rough sets on two universes was firstly defined by Gong and Sun [37]. In [38], the authors focus on the properties of probabilistic rough sets on two universes.

Previous studies are all the extension of Pawlak rough set model on two universes. The equivalence-based information granules in Pawlak rough set are not suitable for dealing with numerical data. It means we have to adopt discretization technique to transform the nonnominal to the nominal for the numerical attributes. The strength of the approach presented in this paper lies in the ability of neighborhood granules to avoid the discretization of the data for the new user and new items problem.

## 3. Preliminary

In this section, we first review the model preference mining in the view of granular computing which was proposed in [4, 39]. Then, the classical neighborhood rough set model on single universe is also reviewed.

### 3.1. Preference Mining in the View of Granular Computing

**Definition 1 (see [40]).**

Knowledge representation is realized via the information system (IS) which is a tabular form, similar to databases. An information system is pair representation realized via the information system (IS) which is a tabular form, similar to databases. An information system is pair IS = (*U*, *A*), where *U* = {*x*_1_, *x*_2_,…, *x*_*n*_} is a nonempty finite set of objects and *A* is a nonempty finite set of attributes.

**Definition 2 (see [40]).**

An equivalence granule can be defined as follows:

(1) $$\begin{array}{c} E\left(\;x\;\right)=\left\{\;\left(\;x,z\;\right)\in U\;|\;\forall a\in A,\;a\left(\;x\;\right)=a\left(\;z\;\right)\;\right\}. \end{array}$$

In an information system, a granule coincides with a concept, which is a basic unit of human thought understood as a pair of intension and extension [41].

The rough sets theory [25], proposed by Pawlak in 1982, is a powerful mathematical method for the study of incomplete or imprecise information. This theory has been successfully applied to many fields, such as data mining, decision making, pattern recognizing, machine learning, and intelligent controlling. The following definitions, which had been defined in the theory of rough set, were employed by Min and Zhu [39].

**Definition 3 (see [42]).**

Let *U* = {*x*_1_, *x*_2_,…, *x*_*n*_} and *W* = {*y*_1_, *y*_2_,…, *y*_*n*_} be two nonempty set of objects. *ℜ*⊆*U* × *W* is the set-valued mapping from universe *U* to *W*. Then, (*U*, *A*, *W*, *B*, *ℜ*) is called the general approximation spaces, where (*U*, *A*) and (*W*, *B*) are two information systems.

This data model can be called two-universe model. In the preference mining method [39], *U* can be consider as the set of users and *W* is the set of items where the two entities are connected by the relation *ℜ*.

**Definition 4 .**

Given the general approximation space (*U*, *A*, *W*, *B*, *ℜ*), for a subset *Y*⊆*W*, we define the lower and upper approximations of *Y* on the space (*U*, *A*, *W*, *B*, *ℜ*) as follows, respectively.

(2) apr_Y=x∈U ∣ Rx⊆Y,apr¯Y=x∈U ∣ Rx∩Y≠⌀.

The above granular computing model is called classical rough set model on two universes.

**Definition 5 (see [39]).**

A granular preference rule is an implication of the form

(3) Ex⟹Eyiff  Rx∩Ey≠⌀,  x∈U,  y∈W.

In the view of rough set [39], the definition of upper approximation is used for describing the preference rules where *E*(*y*) is substituted for the subset *Y*⊆*W*. The granular preference rule can be interpreted that the users in *E*(*x*) like the items in *E*(*y*).

### 3.2. Neighborhood Rough Set on the Single Universe

As the previous sections described, the classical rough set model is unavailable when the datasets are numerical. Hu et al. introduced a neighborhood rough set model in [43] for the heterogeneous data to avoid discretization. The authors considered that the data model is on the single universe. Then, we will give the basic concepts about the neighborhood rough set model on single universe.

**Definition 6 (see [43]).**

*U* is a nonempty finite set of objects and Δ is a given distance function. We say (*U*, *A*, *δ*) is a neighborhood approximation space where

(1) Δ(*x*_*i*_, *x*_*j*_) ≥ 0, if and only if *x*_*i*_ = *x*_*j*_, Δ(*x*_*i*_, *x*_*j*_) = 0;
(2) Δ(*x*_*i*_, *x*_*j*_) = Δ(*x*_*j*_, *x*_*i*_);
(3) Δ(*x*_*i*_, *x*_*k*_) ≤ Δ(*x*_*i*_, *x*_*j*_) + Δ(*x*_*j*_, *x*_*k*_).

**Definition 7 (see [43]).**

Given a neighborhood approximation space (*U*, *A*, *δ*), ∀*x* ∈ *U*, *δ* ≥ 0, we say *δ*(*x*) is a *δ* neighborhood of *x* whose centre is *x* and radius is *δ*, where *δ*(*x*) = {*z*∣Δ(*x*, *y*) ≤ *δ*, *z* ∈ *U*}.

Here, *δ*(*x*) can be seen as the neighborhood granule.

**Remark 8 (see [40]).**

Given two points *x*_*i*_ = {*x*_*i*_^1^, *x*_*i*_^2^,…, *x*_*i*_^*n*^} and *x*_*j*_ = {*x*_*j*_^1^, *x*_*j*_^2^,…, *x*_*j*_^*n*^} in *N*-dimensional Euclidean space, the distance of them can be computed as

(4) Δxi,xj=∑l=1Ndalxi,xj2,dalxi,xj=nom_diffalxi,xjif  al  is  a  nominal  attributenum_diffalxi,xjif  al  is  a  numerical  attribute,

where

(5) nom_diffalxi,xj=0if  xil=xjl1if  xil≠xjl,numdiffalxi,xj=xil−xjlmax⁡Val−min⁡Val.

**Definition 9 (see [43]).**

Given a neighborhood approximation space (*U*, *A*, *δ*), for any subset *X*⊆*U*, we define the lower and upper approximations of *X* on the space (*U*, *A*, *δ*) as follows, respectively.

(6) apr_X=x∈U ∣ δx⊆X,apr¯X=x∈U ∣ δx∩X≠⌀.

Obviously, classical neighborhood rough set model on single universe is not suitable for the user-item data mode structure.

## 4. Data Model and Baseline Evaluation

### 4.1. Data Model

In this study, we illustrate the movie recommendation by making use of MovieLens [44] which is widely used in many preference mining researches (e.g., [4, 10, 39]). We use the version with 943 users and 1,682 movies. Thus, the items are movies in this study. The database schema is follows.

(i) Users: *A* = {u_id, age, gender, occupation}.
(ii) Movies: *B* = {m_id, release − year, genre}.

According to Definitions 1 and 3, *U* and *W* are the set of users and movies. The set *A* and *B* are the features of users and movies, respectively. The specific information can be found in Tables 3 and 4 in Section 7.

The original rating data contains 5 scales showed in Table 1. Reference [4] only considers whether or not a user has rated a movie. That is, it can be established as the mapping relationship *ℜ* from u_13 to m_219 because that u_13 has rated m_219. An example of set-valued mapping *ℜ* is given in Table 2. Then we can mine some preference rules from this type of mapping relationship.

As a matter of fact, 1.0 point means u_13 in all probability dislike m_219. In other words, some of such rules are negative rules. The implication of the word “negative” is “dislike” in this study. Obviously, this type of mapping relationship is unreasonable. Therefore, how to establish the appropriate mapping relationship from users to movies is a key issue for preference mining.

### 4.2. Baseline Evaluation

The rating baseline tends to capture the basic emotions of a user for one item [3]. In this study, it can be considered that a user likes a movie if he (she) gives a higher score than his (her) raging baseline. The simplest computational method of baseline is the overall average rating of movies. For example, the average rating over all movies is 3.7 stars. User Joe can be regarded as an admirer of the movie Avatar if he has given Avatar 4.0 points. However, the authors [3, 45] proposed that the user and item bias exist in the real rating system. Some users, for instance, frequently give higher ratings than others. Similarly, some items always receive higher ratings than others. That is to say, the rating baseline should be personalized. Therefore, we use the baseline evaluation method in [45]. A user's rating baseline for a movie *b*_*ui*_ accounts for the user and item bias effects.

(7) $$\begin{array}{c} b_{ui}=\mu +b_{u}+b_{i}. \end{array}$$

*μ* is the overall average rating. The parameters *b*_*u*_ and *b*_*i*_ indicate the raging bias of user *u* and item *i*, respectively. We also use the example about Avatar to explain the meaning of *b*_*ui*_. Avatar is more popular than an average movie, so it tends to be rated 0.5 stars above the average. On the other hand, Joe is a critical user, who tends to rate 0.1 stars lower than the average. Thus, the baseline estimate for Avatar's rating by Joe would be 4.1 stars by calculating 3.7 − 0.1 + 0.5. It means Joe might not be interested in Avatar if he has given Avatar 4.0 points.

In order to estimate *b*_*u*_ and *b*_*i*_ one can solve the least squares problem [1, 3, 45]:

(8) Cκ=minb∗⁡∑u,i∈κrui−μ−bu−bi2+λ∑ubu2+∑ibi2.

Here, *κ* is all the training samples. The first term ∑_(*u*, *i*)∈*κ*_^ ^(*r*_*ui*_ − *μ* − *b*_*u*_ − *b*_*i*_)^2^ strives to find *b*_*u*_'s and *b*_*i*_'s that fit the given ratings *r*_*ui*_. The regularizing term *λ*(∑_*u*_^^ *b*_*u*_^2^ + ∑_*i*_^^ *b*_*i*_^2^) avoids overfitting by penalizing the magnitudes of the parameters. And then, we can take the derivative with respect to *b*_*u*_ and *b*_*i*_.

(9) ∂C∂bu=−2·bi+2λbu,∂C∂bi=−2·bu+2λbi.

Thus, gradient descent was adopted as learning algorithm [1, 3, 45].

(10) bu⟵bu+αbi−λbu,bi⟵bi+αbu−λbi.

The parameter *α* is set to 0.003 in this study. Thus, *μ*, *b*_*u*_, and *b*_*i*_ can be calculated with the above formulas.

We use this baseline evaluation method for building the mapping relationship between the two universes. A user can be considered as an admirer of a movie if he gives a higher rating than the baseline *b*_*ui*_ of the movie. The fourth column in Table 1 shows the results by the baseline estimate. Then the set-valued mapping *ℜ* is rebuilt in the fourth column in Table 2.

## 5. Neighborhood Rough Set Model on Two Universes

In this section, we build the neighborhood rough set model on two universes (NRSTU).

**Definition 10 .**

Let *U* and *W* be two nonempty finite universes. *δ*(*x*) is a *δ* neighborhood of *x* whose centre is *x* ∈ *U*. *ℜ* is the set-valued mapping from universe *U* to *W* where *ℜ*(*x*)⊆*W*. Then, we have the object set *ℜ*(*δ*(*x*))⊆*W* if *δ*(*x*)⊆*U*. Hence, (*U*, *W*, *ℜ*, *δ*) is called neighborhood approximation space on two universes.

*ℜ*(*δ*(*x*)) is the set of the elements in *W* which are mapped from *δ*(*x*) ⊂ *U*.

**Definition 11 .**

Given a neighborhood approximation space on two universes (*U*, *W*, *ℜ*, *δ*) and *Y*⊆*W*, the lower and upper approximation about *Y* can be defined as

(11) apr_Y=x∈U ∣ Rδx⊆Y,apr¯Y=x∈U ∣ Rδx∩Y≠⌀.

The boundary region, positive region, and negative region can be defined as

(12) BNY=apr¯Y−apr_Y,POSY=apr_Y,NEGY=U−apr¯Y.

**Theorem 12 .**

Let (*U*, *W*, *ℜ*, *δ*) be neighborhood rough sets on two universes. For any *Y*⊆*W*, given two positive numbers *δ*_1_ and *δ*_2_, if *δ*_1_ ≥ *δ*_2_, one has

(13) apr_δ1Y⊆apr_δ2Y,apr¯δ1Y⊇apr¯δ2Y.

**Proof**

(1)  ∀*x* ∈ *U* and *δ*_1_ ≥ *δ*_2_: we have *ℜ*(*δ*_1_(*x*))⊇*ℜ*(*δ*_2_(*x*)). Assuming *R*(*δ*_1_(*x*))⊆*Y*, we have *ℜ*(*δ*_2_(*x*))⊆*Y*. Therefore, we must have $x\in {\underline{apr}}_{\delta_{2}}\left(Y\right)$ if $x\in {\underline{apr}}_{\delta_{1}}\left(Y\right)$. However, *x* is not sure in ${\underline{apr}}_{\delta_{1}}\left(Y\right)$ if we have $x\in {\underline{apr}}_{\delta_{2}}\left(Y\right)$. Hence, ${\underline{apr}}_{\delta_{1}}\left(Y\right)\subseteq {\underline{apr}}_{\delta_{2}}\left(Y\right)$.

(2)  ∀*x* ∈ *U* and *δ*_1_ ≥ *δ*_2_: we have *ℜ*(*δ*_1_(*x*))⊇*ℜ*(*δ*_2_(*x*)). Assuming *ℜ*(*δ*_2_(*x*))∩*Y* ≠ *ϕ*, we have *ℜ*(*δ*_1_(*x*))∩*Y* ≠ *ϕ*. Therefore, we must have $x\in {\underline{apr}}_{\delta_{1}}\left(Y\right)$ if $x\in {\underline{apr}}_{\delta_{2}}\left(Y\right)$. However, *x* is not sure in ${\underline{apr}}_{\delta_{2}}\left(Y\right)$ if we have $x\in {\underline{apr}}_{\delta_{1}}\left(Y\right)$. Hence, ${\bar{apr}}_{\delta_{1}}\left(Y\right)⊇{\bar{apr}}_{\delta_{2}}\left(Y\right)$.

In the practical environment, neighborhood upper approximation is inadequate for describing user preference. For example, we need to know how many items in the granule users like. Here, we proposed a parametric neighborhood rough sets model on two universes where |·| is the cardinality of the set.

**Definition 13 .**

Let (*U*, *W*, *ℜ*, *δ*, *p*) be parametric neighborhood approximation space on two universes. For any 0 ≤ *β* < *ω* ≤ 1, *Y*⊆*W*. Then, the lower and upper approximations of *Y* with *ω* and *β* are as follows.

(14) apr_pωY=x∈U ∣ Rδx∩YY≥ω,apr¯pβY=x∈U ∣ Rδx∩YY>β.

We have proposed a variable precision neighborhood rough sets model in the previous research [46]. In this study, *ω* is the coverage degree of *ℜ*(*δ*(*x*))to *Y*. It does not mean the precision of an approximation. This is why we call this model parametric neighborhood rough sets instead of variable precision neighborhood rough sets.

**Definition 14 .**

Let (*U*, *W*, *ℜ*, *δ*, *p*) be variable precision neighborhood approximation space on two universes. For any *x*_*i*_ ∈ *U*, the neighborhood uncertainty of *x* is defined as

(15) $$\begin{array}{c} {NH}^{x_{i}}\left(\;U,W\;\right)=-\log\frac{\left|R\left(\delta \left(x_{i}\right)\right)\right|}{\left|W\right|}. \end{array}$$

The uncertainty of approximation space is computed as

(16) $$\begin{array}{c} NH\left(\;U,W\;\right)=-\frac{1}{\left|U\right|}\underset{x_{i}}{\sum }\log\frac{\left|R\left(\delta \left(x_{i}\right)\right)\right|}{\left|W\right|}. \end{array}$$

## 6. Preference Rules and Recommendation

In this section, we first define the preference rules by using the neighborhood rough set models on two universes. Then the method of recommendation is also discussed in detail.

### 6.1. Preference Rules

Min has given the formulation of the preference rule on the view of equivalence granule [4]. In our study, we define a neighborhood granular preference rule by extending Min's work.

**Definition 15 .**

Given a neighborhood approximation space on two universes (*U*, *W*, *ℜ*, *δ*) and *x*_*i*_ ∈ *U*, *y*_*j*_ ∈ *W*. A neighborhood granular preference rule can be described as follows.

(17) $$\begin{array}{c} \delta \left(\;x_{i}\;\right)⟹\delta \left(\;y_{j}\;\right)\;\text{iff }R\left(\delta \left(x_{i}\right)\right)\cap \delta \left(y_{j}\right)\neq ⌀. \end{array}$$

The formulation is consistent with the upper approximation as Definition 11. In an information system, a granule is a basic unit of human thought [41]. In a regular recommended system, such as CF method, the meaning of neighborhood is that the similar people have similar interests [45]. Therefore, the definition of neighborhood upper approximation can be interpreted that some similar users in *δ*(*x*_*i*_) like the similar items in *δ*(*y*_*j*_).

The rules are usually evaluated through two measures, namely, support degree and confidence degree, which are well defined in [47] for the single universe model. In the two-universe model, there are user and item support degree of the rules, respectively.

(18) supuser=δxiU≥η,supitem=δyjW≥γ.

On the other hand, the higher proportion of items in *δ*(*y*_*j*_) users like also indicates that the preference rule is stronger. Hence, we also use the third measure called confidence degree for mining the stronger rules.

(19) $$\begin{array}{c} conf=\frac{\left|R\left(\delta \left(x_{i}\right)\right)\cap \delta \left(y_{j}\right)\right|}{\left|\delta \left(y_{j}\right)\right|}\geq \omega . \end{array}$$

Thus, the parameter *ω* in the parametric neighborhood rough sets model can serve as the confidence degree of the rules. This is why we propose the parametric neighborhood rough sets model in Definition 13. The formulation of preference rules is redefined by using the lower approximation in the parametric neighborhood rough sets model.

(20) $$\begin{array}{c} \delta \left(\;x_{i}\;\right)⟹\delta \left(\;y_{j}\;\right)\;\text{iff }\frac{R\left(\delta \left(x_{i}\right)\right)\cap \delta \left(y_{j}\right)}{\delta \left(y_{j}\right)}\geq \omega . \end{array}$$

Here, the neighborhood lower approximation is employed for the definition of the preference rule. This type of rule can be read as “the users in *δ*(*x*_*i*_) like at least *ω*% of items in *δ*(*y*_*j*_)”. In our study, we only consider the condition that the users and items are described by all of the features.

A straightforward algorithm for preference rules mining is given by Algorithm 1 which has two steps.

**Step 1 .**

Search all neighborhood granules meeting the minimal support threshold of user granule *η* and *γ*. This step corresponds to Lines 1 and 2 of the algorithm, where *G*_user_ and *G*_item_ stand for user granules and item granules, respectively.

**Step 2 .**

Check all possible rules regarding *G*_user_ and *G*_item_ and output valid ones. This step corresponds to Line 3 through Line 9 of the algorithm.

### 6.2. Preference Rules for Recommendation

The recommendation method is derived from the idea that the neighbors have the same taste. Given a preference rule *δ*(*x*_*i*_)⇒*δ*(*y*_*j*_) and a new user *u*, we recommend items in *δ*(*y*_*j*_) if *u* is a neighbor of *x*_*i*_.

The performance of a recommender is evaluated mainly by the recommendation accuracy [4]. Formally, let the number of recommended items be *M* and the number of appropriate recommendations *N*; the accuracy is *N*/*M* [4]. In our study, appropriate recommendation can be interpreted that the real score of the recommended item is higher than the rating baseline of the user. We will elaborate it in detail by the next experiments.

## 7. Experiments

In this section, we will evaluate our model through experimentation. In this study, the method of preference mining has improved by two aspects as follows.

1. NRSTU is proposed to overcome the cold-start problem.
2. NRSTU enhanced the effectiveness of preference mining by avoiding the discretization and the dividing positive rules and negative rules.

We design three experiments to verify the two points above. First of all, we give an experimental example to show the details of NRSTU-based preference mining. It elaborates how to solve the cold-start problem by using NRSTU. Then, we will discuss the parameters of our model through experimental analysis. To compare the effectiveness of NRSTU, we choose the classical rough set model on two universes (RSTU) as the benchmark. RSTU are employed in [4, 39] for preference mining. We download the discretization samples from [48] which are preprocessed by Min as in [39]. This experiment is called NRSTU versus RSTU.

### 7.1. The Meaningfulness of Neighborhood Preference Rules and Recommendation

Firstly, we look at some rules mined from MovieLens dataset by Algorithm 1. The setting is as follows: *η* = *γ* = 0.02 and *ω* = 0.3. The training samples percentage and test samples percentage are 90% and 10%, respectively. We can get lots of positive rules form the users who are labeled “like the movies.” Similarly, some negative rules are also obtained from the users who dislike those movies. Some of them are listed below as the examples.

Some positive preference rules are as follows:

(1) *δ*_user_(13)⇒*δ*_movie_(1641).
(2) *δ*_user_(32)⇒*δ*_movie_(1680).
(3) *δ*_user_(44)⇒*δ*_movie_(1165).
(4) *δ*_user_(940)⇒*δ*_movie_(861).

Some negative preference rules are as follows:

(5) δuser937  ⇒  δmovie861.
(6) δuser936  ⇒  δmovie1140.
(7) δuser940  ⇒  δmovie192.

As in Tables 3 and 4, we use 8 movies and 7 users to expound the meaningfulness of neighborhood preference rules. For example, *δ*_user_(32)⇒*δ*_movie_(1680) means that the users in *δ*_user_(32) like the movies in *δ*_movie_(1680). It can be interpreted that the female students who are about 28 years old like the movies which are labeled drama and romance. Then, we can recommend movies to other users by using the neighborhood preference rule as the Algorithm 2. Suppose u_230 is a new user who has not rated any of the movies. The classical CF-based method cannot work if we do not know any of the historical rating data of u_230. We can get her registration information. Then we will know she is a female student. Hence, we can recommend the movie m_280 to her because of m_280 ∈*δ*_movie_(1680). As a matter of fact, m_280 is scored 4.0 points by user u_230 where the baseline of u_230 on movie 280 is 2.8 (see Table 1). It means that user u_230 really enjoys the movie m_280. Here we say it is an appropriate recommendation.

On the other hand, our method also mines some negative preference rules which can solve the problem in [4, 39]. The authors only consider whether or not a user has rated a movie. A user will be labeled “like” if he has rated a movie. It means that the user, who gives a movie low mark, could be regarded as an admirer of the movie. That is to say, the negative preference rules (5), (6), and (7) will be regarded as the positive preference rules in the view of [4, 39]. For example, as Table 1 in Section 3, suppose u_13 is a new user who is a neighbor of u_937. Movie m_219 will be recommended to u_13 because of m_219 ∈*δ*_movie_(861). As a matter of fact, m_219 is scored 1.0 point by user u_13 where the baseline of u_13 on movie 219 is 2.3. It means that user u_13, who is a male educator and 47 years old, does not like the horror movie at all. Apparently it is an improper recommendation. This experiment shows the baseline evaluation in an important step in preference mining

### 7.2. Parameters Discussion

There are four parameters in our model. They are neighborhood metric *δ*, confidence degree *ω*, support degree *η*, and *γ*, respectively. Literature [43] has explained that the result is optimal if threshold *δ* is set between 0.1 and 0.2 in the neighborhood system. In our study, threshold *δ* is set to 0.15. Then, the selection of *ω*, *η*, and *γ* is discussed through a series of experiments. We set *η* = *γ* from 0.02 to 0.12 with step 0.02 because we cannot get any of rules when *η* = *γ* > 0.12. We try confidence degree *ω* from 0.05 to 1.0 with step 0.05. For the MovieLens datasets, we randomly divide the samples into 10 subsets and use nine of them as training set and the rest one as the test set. After 10 rounds, we compute the average recommendation accuracy as the final performance.

**Figure 1:**
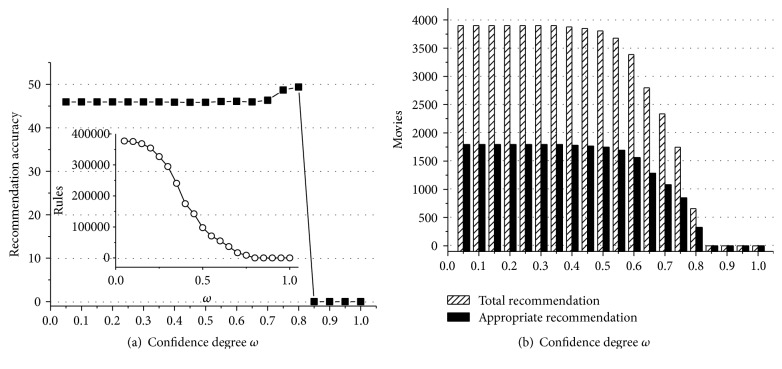
The results of recommendation where the support degree is *η* = *γ* = 0.02. (a) Recommendation accuracy (%) and amount of the rules with the confidence degree *ω*; (b) total and appropriate recommendations with the confidence degree *ω*.

**Figure 2 fig2:**
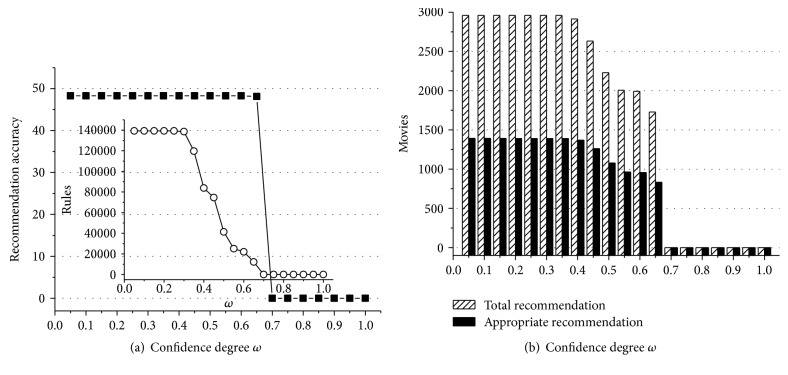
The results of recommendation where the support degree is *η* = *γ* = 0.04. (a) Recommendation accuracy (%) and amount of the rules with the confidence degree *ω*; (b) total and appropriate recommendations with the confidence degree *ω*.

**Figure 3 fig3:**
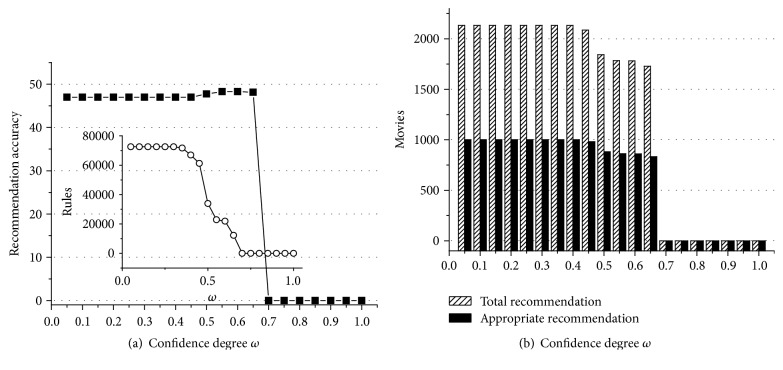
The results of recommendation where the support degree is *η* = *γ* = 0.06. (a) Recommendation accuracy (%) and amount of the rules with the confidence degree *ω*; (b) total and appropriate recommendations with the confidence degree *ω*.

**Figure 4 fig4:**
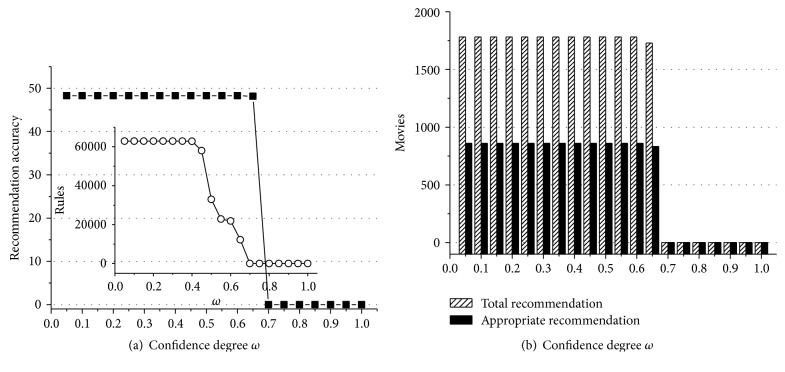
The results of recommendation where the support degree is *η* = *γ* = 0.08. (a) Recommendation accuracy (%) and amount of the rules with the confidence degree *ω*; (b) total and appropriate recommendations with the confidence degree *ω*.

**Figure 5 fig5:**
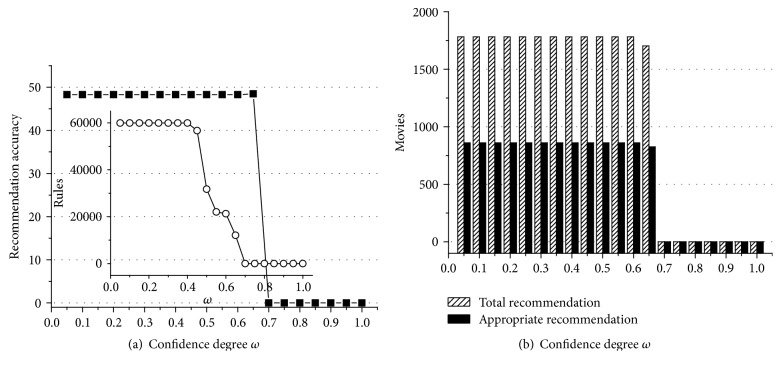
The results of recommendation where the support degree is *η* = *γ* = 0.10. (a) Recommendation accuracy (%) and amount of the rules with the confidence degree *ω*; (b) total and appropriate recommendations with the confidence degree *ω*.

**Figure 6:**
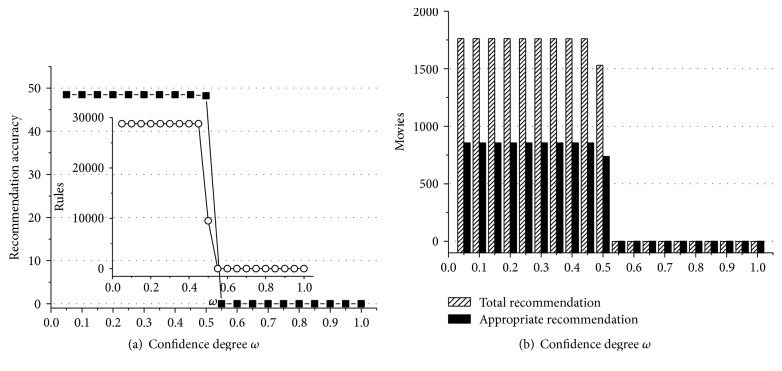
The results of recommendation where the support degree is *η* = *γ* = 0.12. (a) Recommendation accuracy (%) and amount of the rules with the confidence degree *ω*; (b) total and appropriate recommendations with the confidence degree *ω*.

According to experiment data above, we can obtain some useful conclusions. First of all, Figures 1 [Fig fig2] [Fig fig3] [Fig fig4] [Fig fig5]–6 show that the highest recommendation accuracy always keeps from 45% to 50% if 0 < *ω* ≤ 0.55 in most cases. When we increase the numerical value of *ω* from 0.45 to 0.55, it concomitantly reduces the number of the rules. However, total and appropriate recommendations do not significantly reduce in this interval. The amount of the rules will drop to 0% if *ω* ≥ 0.65. Then there are no longer any of recommendations. That is, we can obtain the highest recommendation accuracy by fewer rules when 0.45 < *ω* ≤ 0.55. On the other hand, the amount of total and appropriate recommendations did not significantly reduce when 0.45 < *ω* ≤ 0.55 in most cases. It can be concluded that some preference rules are redundant if *ω* is set in (0, 0.45]. As a matter of fact, we just need more recommendations and the highest recommendation accuracy by using the minimum amount of rules. In this point of view, 0.45 < *ω* ≤ 0.55 is a cost-effective choice for confidence degree *ω*.

For the support degree *η* and *γ*, they only impact on the amount of the rules and recommendations showed in Figures 1(b), 2(b), 3(b), 4(b), 5(b), and 6(b). The recommendation accuracy has nothing to do with *η* and *γ*. It is obvious that the higher the support degree we set the less rules and recommendations we can get. Nonetheless, the high support degree means the strong preference rules. Consequently, it is more reasonable to set the support degree based on actual demand. It depends on which one is more needful between stronger preference rules and more recommendations for a real recommended system. We can even set different values for *η* and *γ* on a per-destination basis. Hence, it becomes an open-ended question.

### 7.3. NRSTU versus RSTU

In this section, we choose RSTU [4] as the benchmark. In [4], the authors proposed a cold-start recommendation approach by using the classical parametric rough set model. The main difference between NRSTU and RSTU is the granules that are structured by the equivalence relation in [4] rather than neighborhood relation. It means that the users (or items) can be sorted into one granule if their features are identical. Actually, the features are rarely exactly alike because of the numerical features such as age and release year. Therefore, it has to adopt discretization technique to transform the nonnominal to the nominal, which would bring loss of information inevitably. In this experiment, the setting is as follows: *η* = *γ* = 0.02 and *ω* = 0.5.

The random recommender, which has an accuracy close to 6.2%, is illustrated for comparison in [4]. As is shown in Table 5, NRSTU yield better performance than RSTU and random recommender obviously. In particular, NRSTU improves the recommendation accuracy by about 19%. There are two reasons for the explanation. The first reason is that RSTU is unreasonable to measure similarity or dissimilarity with Euclidean distance as to categorical attributes in numerical methods. Hu et al. [43] pointed out that there are at least two categories of structures lost in discretization: neighborhood structure and order structure in real spaces. For example, we know the distances between samples and we can get how the samples are close to each other in real spaces. NRSTU addresses this deficiency by the neighborhood granule structure. Furthermore, there is no distinction between positive rules and negative rules in [4]. It is consequent to give some unreasonable recommendations by RSTU which has been discussed in the first experiment. Thus it can be seen that NRSTU is more effective for dealing with the problem of preference mining.

## 8. Conclusion and Future Work

In this study, we proposed the parametric neighborhood rough set model on two universes for dealing the problem of preference mining. Firstly, baseline evaluation is investigated for dividing the positive and negative mapping. Furthermore, the definition of neighborhood lower approximation is proposed to define the user preference rules. The algorithms about preference rules mining and item recommendation are also given. Experiment 1 elaborates how NRSTU can overcome the cold-start problem. It simultaneously shows that baseline evaluation can avoid some unreasonable recommendations. The parameters of NRSTU are discussed in detail in Experiment 2. The last experiment shows that NRSTU improves the recommendation accuracy by about 19%. It can be concluded that NRSTU is more effective for dealing with the problem of preference mining.

The future work could move along two directions. First, many other rough set models also can be used to describe the user preference rules, such as kernel rough set and fuzzy rough set. Comparative analysis about the effectiveness of these rough set modes for preference mining is an important issue. Second, the application of our model for dealing with big data is necessary. Consequently, the version of NRSTU within distribute framework requires further attention.

## Figures and Tables

**Algorithm 1:**
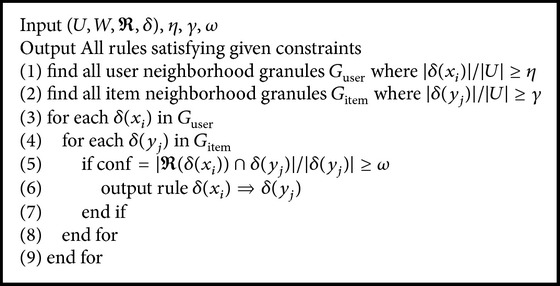
Preference rules mining.

**Algorithm 2:**
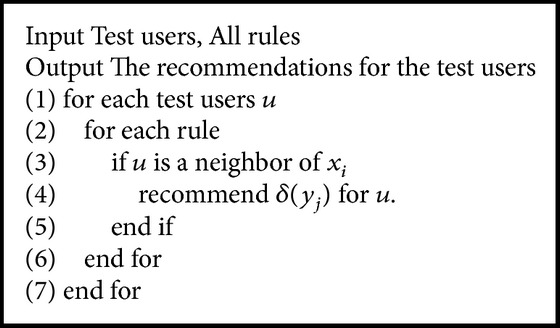
Recommendation method.

**Table 1:** Rating table and baseline evaluation.

Users	Movies	Ratings	Baseline
u_230	m_280	4.0	2.8
u_13	m_219	1.0	2.3

**Table 2:** Rating table and baseline evaluation.

Users	Movies	Ref [4]	Our study
u_230	m_280	1	1
u_13	m_219	1	0

**Table 3:** Information of movies.

ID	Release year	Genre
m_192	1980	Drama
m_861	1986	Horror
m_1140	1994	Comedy
m_1165	1996	Comedy, drama
m_1641	1995	Documentary
m_1680	1998	Drama, romance
m_280	1996	Drama, romance
m_219	1984	Horror

**Table 4:** Information of users.

ID	Age	Gender	Occupation
u_13	47	Male	Educator
u_32	28	Female	Student
u_44	26	Male	Technician
u_936	26	Male	Other
u_937	48	Male	Educator
u_940	32	Male	Administrator
u_230	28	Female	Student

**Table 5:** The comparison between NRSTU and RSTU.

	NRSTU	RSTU
Rules	97508.6	14260.2
Total recommendation	3804.7	854.1
Appropriate recommendation	1744.6	223.6
Recommendation accuracy	45.85%	26.17%
